# We Need to Explore Alternatives to the ASI: Response to Commentaries

**DOI:** 10.1177/14550725261506178

**Published:** 2026-11-12

**Authors:** Alexander Bjork

**Affiliations:** 1 Department of Social Work, 376168 Stockholm University, Stockholm, Sweden

Having read the three commentaries on my debate article (Bramnes, 2026; Kuuisto, 2026; Nielsen, 2026), I am pleased to note that none of the authors challenges my central argument concerning the limited evidence supporting the clinical use of the Addiction Severity Index (ASI) (Björk, 2026). There are, however, comments on adjacent issues that I wish to address in this response. These concern: (1) the multiple purposes of the ASI; (2) the unclarities regarding norm data for the ASI; (3) the benefits of standardized assessments in general; (4) what counts as evidence for assessment methods; and (5) how to explain the poor usage of the ASI. I will go through these points one by one.

1. In the original debate article, I pointed at the unclear link between evidence and recommendation in the Swedish National Board of Health and Welfare's national clinical practice guidelines (National Board of Health & Welfare (NBHW), 2019). Given that these guidelines focus on the ASI as a clinical tool (NBHW, 2019, p. 27), my argument centered on this aspect alone. As Nielsen (2026) and Kuuisto (2026) point out in their comments, the ASI has multiple purposes and intended users. In addition to clinical use, the ASI can also be used as outcome measure in treatment research and for monitoring aggregate client outcomes. In this respect, I agree with Nielsen (2026) that the ASI is unique in combining these different purposes. Indeed, this may to some degree explain the inexplicably strong recommendation of the ASI in the Swedish guidelines.

In this context, it is however worth noting that the ASI's limited clinical utility also undermines its monitoring purposes. The ASI data used in outcome monitoring are often collected by social workers in their everyday work. The limited use of the ASI in social service practice therefore means that the monitoring data do not sufficiently represent the clinical population. This is particularly evident in the failed attempts to produce norm data (Armelius et al., 2009) mentioned in my original debate article. In sum, the multiple purposes of the ASI make sense in theory, but in practice, it seems to deliver mainly on its research purpose.

2. When it comes to the issue of norm data for the ASI, I believe Nielsen (2026) misunderstands my point. Contrary to what Nielsen (2026) implies, I do not argue in favor of any form of norm data, whether based on normal or clinical populations. My argument is simply that the Swedish guideline panel does not adhere to its own criteria and that the alleged norm data fall short of common scientific standards.

3. In defending the ASI, Nielsen (2026) highlights the benefits of standardized assessments in general. I agree that there may be several benefits with a structured approach to assessment: reducing unwarranted variation and different kinds of biases and providing a checklist to ensure that important areas of assessment are not overlooked (Björk, 2025). This also relates to Bramnes (2026) comment that the ASI can function as a benchmark. My critique, however, was not directed toward standardized assessments in general, but toward the ASI specifically. It is quite clear from research on the use of the ASI that social workers find it too comprehensive, with too many detailed questions.

I am convinced that it is possible and necessary to think about standardized assessments in a different way, at least for the social services. The method that we are developing in the FUSS (Ekendahl, 2023–ongoing) research program can serve as an example. Our ambition is to find a better balance between standardization and flexibility so that the assessment method can safeguard against subjectivity at the same time as allowing necessary flexibility for clients and professionals. Similar to the ASI, the method is structured around 10 question areas, and each area contains instructions not only on important aspect to cover, but also possible open-ended question to ask the client. Each question area contains at least one client rating question that can be used in client outcome monitoring (exactly what Nielsen (2026) hoped in her comment). Regardless of how promising this method turns out to be, I think social work professionals deserve a better standardized assessment method and benchmark than they currently have.

4. It is clear from the comments to the original debate article that there is a need to discuss what should be counted as practice-relevant evidence for assessment methods, a discussion that also includes methodological aspects. The commentators and I agree that aspects of validity and reliability do not constitute appropriate evidence for the clinical usefulness of assessment methods, even though Nielsen (2026) mentions that reliability studies can be good opportunities to learn a method (but that is beside the point here). We also agree that evidence related to assessment methods should focus on client outcomes, or at least what happens after assessment (as Kuuisto (2026) proposes in her comment). While Nielsen (2026) opposes some of my arguments, I believe that our overall positions may be closer than they appear.

In the original debate article, I use the Grading of Recommendations, Assessment, Development and Evaluation (GRADE) handbook (GRADE, 2026) as an argument for what should be counted as appropriate evidence. Nielsen (2026) suggests that GRADE is not applicable for the discussion of assessment methods and incorrectly claims that GRADE is only used for intervention studies. There is, in fact, an entire chapter on diagnostic tests and strategies in the GRADE handbook (chapter 7), which I believe is useful when discussing what counts as appropriate evidence for assessment methods. In line with GRADE, I suggest that so called diagnostic intervention studies entail proper research designs for providing practice-relevant evidence for assessment methods. This is because this research design makes it possible to study whether one assessment method leads to better client outcomes than another.

Nielsen (2026) also objects to the relevance of diagnostic interventions studies since the ASI is not a diagnostic test. Even though it is correct that the ASI is not a diagnostic test (which I also note in my debate article), I think Nielsen misses my overall point. Similar to diagnostic tests, the overall purpose of assessment methods is to support professionals in making the best decisions about interventions for their clients. And better decisions about interventions may lead to improved client outcomes. This is why diagnostic intervention studies can be relevant also when it comes to assessment methods. Obviously, we should not call it diagnostic intervention studies, but I maintain that the design and underlying principles are applicable in the study of assessment methods.

Indeed, this research design is in line with Nielsen's own study which she cites as evidence for the ASI. Based on Nielsen and Nielsen (2018), she claims that “we know that implementing structured assessment interviews like ASI and using the collected data during treatment, improves outcome of treatment.” The mentioned study compares two client cohorts over time and studies whether a matching procedure based on ASI assessment and an additional motivational session will improve client outcomes compared with treatment as usual. While this study uses a research design that shares the underlying principles of diagnostic intervention studies that I call for, this specific study cannot be used as evidence for the ASI the way Nielsen claims. This is because the ASI was used in the assessment of both cohorts and because the improvements in client outcomes could just as easily have been caused by the additional motivational session rather than by the matching procedure. If the study had compared the ASI with another assessment method and found better client outcomes in the ASI cohort, it would undoubtedly constitute evidence in favor of the ASI.

5. I have one final short remark on Nielsen's (2026) comment that low usage of the ASI does not necessarily equate poor usability. I can agree with this comment to some extent. The explanation of the low usage of the ASI can roughly be divided into two categories: the ASI itself and the *implementation* of the ASI. The implementation of the ASI in Sweden has been ongoing for almost two decades and has been backed with substantial governmental and municipal support. Despite this, the implementation results have been quite poor in terms of usage. With this in mind, I find it fairly uncontroversial to claim that the explanation of the low usage can be attributed to the ASI itself and its poor usability.

I have conducted research on the ASI on and off for about 15 years and have noticed its shortcomings for quite some time. I would therefore not say that my critique reflects the kind of first impulse to find faults on old solutions that Bramnes (2026) cautions against in his comment. I believe the ASI has now had a fair trial in Sweden. Combined with the limited evidence of its superiority over alternative approaches, this gives us good reason to explore other options.
